# Relationship of phase angle and peak torque of knee extensors with the performance in six-minute step test in haemodialysis patients

**DOI:** 10.1186/s12882-021-02256-7

**Published:** 2021-02-08

**Authors:** Camila Porto Brito, Igor Gutierrez Moraes, Cláudio Luders, Christina May Moran de Brito, Wellington Pereira Yamaguti

**Affiliations:** 1 Hospital Sírio-Libanês, Rehabilitation Centre, Dona Adma Jafet Street, 91 – Bela Vista, São Paulo, SP 01308-050 Brazil; 2 Hospital Sírio-Libanês, Haemodialysis Centre, Dona Adma Jafet, 91 - Bela Vista, São Paulo, SP 01308-050 Brazil

**Keywords:** Chronic kidney disease, Exercise tolerance, Peripheral muscle function, Phase angle, Haemodialysis

## Abstract

**Background:**

Chronic kidney disease is a systemic disease affecting not only renal function, but also endocrine, cardiovascular, and skeletal muscle systems, with broad impact on functionality. Therefore, the assessment of body composition, peripheral muscle function, and exercise tolerance is also of great importance in this population. In addition, the development of reliable strategies that use feasible tools in clinical practice is of great importance in the functional diagnosis, clinical management and prescription of physical activity.

**Objective:**

To assess the relationship between phase angle and peak torque of knee extensors with the performance in six-minute step test of patients with chronic kidney disease undergoing haemodialysis.

**Methods:**

Observational and cross-sectional study at Haemodialysis Centre of Sírio-Libanês Hospital. The evaluations took place 3 days before the haemodialysis sessions. On the first day, patients underwent anthropometric evaluation and biochemical tests (lactate, urea, creatinine, albumin, ferritin and growth factor similar to insulin type 1 [IGF1]). On the second day, performed electrical bioimpedance and exercise tolerance assessed using the six-minute step test. On the last day, the evaluation of knee extensor muscles strength using isokinetic dynamometry was performed.

**Results:**

Eighteen subjects were included, with a mean age of 62.3 ± 17.6 years. A positive linear correlation was observed between the phase angle, derived from the electrical bioimpedance, and the performance in six-minute step test in these patients. It was also possible to observe a linear correlation between phase angle and IGF1, handgrip strength, peak torque of knee extensors and quality of life questionnaire. The peak torque of knee extensors also presented a linear correlation with IGF1, the performance in six-minute step test and mal-nutrition and inflammation score.

**Conclusion:**

Phase angle and peak torque of knee extensors were correlated with the performance in six-minute step test. Therefore, the phase angle seems to be an important marker of reduced peripheral muscle function and exercise tolerance in haemodialysis patients.

**Supplementary Information:**

The online version contains supplementary material available at 10.1186/s12882-021-02256-7.

## Background

Chronic kidney disease (CKD) is a multi-factorial syndrome that affects not only renal function, but also cardiovascular, respiratory, endocrine and musculoskeletal functions [1]. In advanced stages, hydroelectrolytic imbalance occurs, culminating in systemic changes in CKD: metabolic acidosis, systemic inflammation, anaemia, oedema and uraemia, causing major changes in body composition and peripheral muscle function [2]. At this point, sometimes it is necessary to initiate kidney replacement therapy (KRT), which tends to increase life expectancy in this population; however, individuals in KRT commonly present worsening of peripheral muscle function, reduced cellular integrity, reduced exercise tolerance, and worse quality of life [3, 4]. Uremic sarcopenia and protein- wasting energy are common findings in these individuals, both of which are associated with mortality, CKD progression and worse prognosis in this population [5–7].

Evaluation of body composition in this population using electrical bioimpedance (BIA) is effective, the phase angle (PA) derived from BIA, which is commonly reduced in this population, appears to be a strong marker of reduction of peripheral muscle function, exercise capacity and mortality [8, 9]. Other data demonstrate that peripheral muscle strength and functional capacity appear to be reduced in patients with CKD on haemodialysis (HD) treatment [10]. These reductions in this population also appear to be caused by a reduction in muscle electrical activity and time on HD [11].

Most of the available studies, correlated PA with mobility tests, however, mobility tests are not able to assess exercise tolerance, so their use and interpretation of results is limited [12]. Another author used the six-minute walk test (6MWT) to in elderly, nevertheless, the 6MWT requires specific physical space and that is sometimes unavailable, especially in the clinical and hospital context [13]. In contrast, instruments such as the six-minute step test (6MST) are able to assess exercise tolerance, without the need for ample physical space [14]. In addition, studies using the 6MST have observed that the physiological behaviour of individuals during this test seems to be closer to the cardiopulmonary test then 6MWT [15–17].

Therefore, studies that encourage the use of viable instruments for assessing exercise tolerance of HD patients and also that find new instrument options for simple assessments that can predict the exercise tolerance of these individuals are necessary. So far, we do not know of any study that has used the 6MST to assess exercise tolerance in this population and correlated its performance with the PA and peak torque of knee extensors. Therefore, the aim of this study was to evaluate the correlation between PA and peak torque of knee extensors with the performance in 6MST of CKD patients undergoing HD.

## Methods

This is a cross-sectional study that aims to assess the relationship of PA and peak torque of knee extensors with the performance in 6MST of patients with CKD undergoing HD. The sample was obtained consecutively at the HD Centre of the Hospital Sírio-Libanês, located in the city of São Paulo-SP-Brazil. The following inclusion criteria were considered: 1) patients with CKD undergoing diurnal HD; 2) patients older than 18 years old; 3) without pacemaker; 4) without cognitive or motor deficits that make evaluations unfeasible; and 5) practice regular physical activity less than twice a week. Exclusion criteria were as follow: 1) inability to perform assessments within technical acceptability criteria; and 2) cardiorespiratory instability during the tests. The study was previously approved by the Research Ethics Committee of the Sírio-Libanês Hospital (approval protocol number 2017–95), and written informed consent was provided by all participants. This study also complies with the standards of the Helsinki Declaration [18].

### Study design and experimental procedures

All participants in the current study underwent an alternately three-day evaluation protocol, before the HD session. At the beginning of the assessments, personal and health information was collected from all participating individuals. The structure form and other questionnaires are available in the section of supplementary materials (file 2).

### Evaluated parameters

#### Biochemical markers

Collection of venous blood carried out by the sector’s nursing team, for further laboratory analysis of: urea, creatinine, IGF1, lactate, ferritin and albumin. The blood sample was taken from the HD catheter or fistula, therefore, there was no need for a new puncture for blood collection. The HD efficiency coefficient was calculated for all the patients according to Kidney Disease: Improving Global Outcomes (KDIGO) [19] recommendations.

#### Body composition and cellular integrity

Electrical bioimpedance (Fresenius Medical Care, Wanchai, Hong Kong) was performed to evaluate body composition. Two self-adhesive patches were applied to the back of the hand and the foot, on the same sides, after cleaning the area with an alcoholic solution. To measure the cellular integrity we used PA values. After the measurement, with 50 Hz frequency, we assess the data using Fresenius Medical Care software [20], to calculate the predicted values, considering gender and age, with the use of Barbosa-Silva et al. equation [21].

#### Body nutrition and inflammation

To measure the body nutrition and inflammation the Malnutrition and Inflammation Score (MIS) was applied. The score consists of ten items regarding clinical history, functional capacity, biochemical parameters and anamnesis. This instrument is a quantitative tool, with good reproducibility, capable of providing data, even in the face of slight changes [22, 23].

#### Peripheral muscle function

Peripheral muscle function was assessed using three tools, as follows. The first one was the Medical Research Council (MRC) score, which consists of the assessment of six specific bilateral movements of upper and lower limbs [24]. Then, handgrip strength (HGS) through of a handheld hydraulic dynamometer (SAEHAN corporation; Yangdeok-Dong, South Korea) following the protocol recommended by the American Society of Hand Therapists [25]. The best mark among three acceptable evaluations was considered as the measure of HGS. The rest period between measurements was 1 min. For the assessment of HGS, the dominant limb was evaluated, as long as there was no fistula. To calculate the reference values, the equations of Novaes et al. were used [26]. And we also used isokinect dynamometry which is the gold standard method to assess peripheral muscle strength. Isokinetic dynamometry (Biodex System III, Biodex Medical Systems, Shirley, USA) of knee extensors was performed. During the tests, the individual sat upright on the dynamometer chair, with back support, stabilized with belts at the level of the thorax and pelvis. The protocol consisted of a series of knee extension repetitions [27, 28]. The first series had five repetitions at an angular speed of 60°; the second series had 10 repetitions at an angular speed of 180°; and the last series had 30 repetitions at an angular speed of 240°. The series had 1 min of rest between them [28, 29]. Maximum isokinetic strength was defined as the highest peak torque in the series of five repetitions and 60 degrees of angular velocity. The reference equation of predicted values was proposed by Neder et al. [30] To determine the fatigue index, the average peak torque variation over 30 repetitions was calculated. The mean peak torque of the first five repetitions and the mean peak torque of the last five repetitions were the measure, subsequently, the mean variation between both was analysed. A higher proportional decline in peak torque in the last five repetitions seems to be correlated with muscle fatigue. The isokinetic evaluation was performed on both limbs; however, data from the dominant limb were used for the analysis [31, 32].

#### Exercise tolerance

Exercise tolerance was measured using the six-minute step test (6MST), which is an applicable and reliable field test [33]. The step was positioned to ensure that there was no displacement during the test, and the patient was instructed to go up and down the step, with the leg that felt most comfortable, as fast as possible. Blood pressure, peripheral oxygen saturation, heart rate, dyspnoea, and lower limb fatigue were measured in three timepoints: before the start of the test, at the end and after 2 min of recovery [34]. To measure the dyspnoea and lower limb fatigue the Modified Borg Scale was used [35]. The patients received incentive phrases every minute. The test could be discontinued if there was intense dyspnoea, fatigue, or any other severe discomfort, and participants were encouraged to retake the test as soon as possible without the timer being paused. To calculate the predicted step values, the equation proposed by Arcuri et al. was used, with the adjustment for age and gender of the individuals [36].

#### Quality of life

Quality of life was assessed with the Kidney Disease Quality of Life Short form (KDQOL-SF), this questionnaire is valid, adapted and reliable for the Brazilian population [36]. It is a specific disease measure that assesses patients’ perception of the influence of CKD on physical, socioeconomic and physiological aspects. The questionnaire contains 80 items divided into specific domains [37]. In this study, we chose to use the score of the physical and emotional perception domain, taking into account that this domain appears to be correlated with functional capacity and peripheral muscle strength [36-39].

### Statistical analyses

For statistical analysis, the Prism 8 (GraphPad, San Diego, USA) was used. Data are presented as mean ± SD. The Shapiro–Wilk test was used to evaluate the normality of the data. To assess the relationship between variables, a linear regression was performed. According to the COSMIN guidelines, it is possible to perform the sample size calculations for expected correlations among measures [40]. Sample size was calculated expecting to find a moderate correlation between functional capacity and peripheral muscle strength by considering the following assumptions: 0.6 correlation coefficient, a desired power of 0.8, and an alpha value of 0.05. The ratio coefficient used for the sample calculation was based on that of the study by Bruggeman et al. [41], who found a correlation of 0.6 between peripheral muscle function and functional capacity of individuals undergoing HD. Therefore, the need for 17 patients to be included in the study was determined. For all tests, *p* < 0.05 was considered statistically significant.

## Results

### Sample characterization

**Fig. 1:**
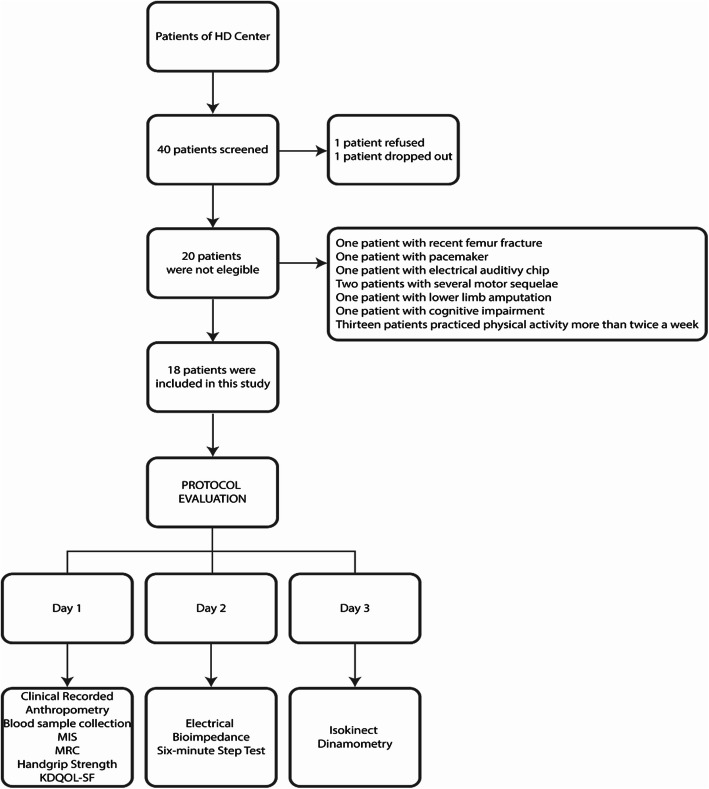
Study flowchart. Abbreviations: MIS, Malnutrition and inflammation score; MRC, medical research council; KDQOL-SF, Kidney Disease Quality of Life-ShortForm; HD, Haemodialysis

A total of 40 patients were screened, but only 20 met the inclusion criteria. Of these 20 subjects, one refused and one dropped out during the evaluations (Fig. 1). The main reason for non-inclusion was the practice of regular physical activity more than twice a week. Thus, eighteen subjects were included (14 males), with a mean age of 62.3 ± 17.6 years.

Body mass index was normal in 44.5% of the patients and only 16.6% were obese. Comorbidities were collected from the patients during anamnesis. Most common comorbidities in these individuals were arterial systemic hypertension and diabetes mellitus.

The baseline characteristics are described in Table 1.

**Table 1:** Characterization of the study participants

**Demographic and anthropometric characteristic**	
Gender Male/Female (n)	14 / 4
Age (years)	62.3 ± 17.6
Body Mass (kg)	81.4 ± 15.9
Height (cm)	172.3 ± 7.5
BMI (kg/m^2^)	27.6 ± 4.6
Lean Mass (kg)	13.1 ± 2.0
Fat Mass (kg)	15.6 ± 5.0
Months undergoing HD (n)	14.2 ± 7.8
**Presence of comorbidities (%)**	
Arterial Systemic Hypertension	66.6
Diabetes Mellitus	61.1
Dyslipidaemia	55.5
Coronary Insufficiency	50.0
Acute Myocardial Infarction	38.8
Stroke	11.1
COPD	5.5
**Biochemical tests**	
KtV(ec)	1.2 ± 0.4
Creatinine(mg/dL)	9.7 ± 3.6
Urea (mg/dL)	161.7 ± 34.4
Albumin (mg/dL)	3.9 ± 0.3
Ferritin (mg/dL)	387.7 ± 183.1
Lactate (mg/dL)	10.9 ± 5.2
IGF1 (mg/dL)	201.0 ± 53.6

Notes: Data are presented as mean ± SD. *Abbreviations*: *kg* Kilogram, *cm* Centimetres, *BMI* Body mass index, *m* Meter, *n* Number of months, *COPD* Chronic obstructive pulmonary disease, *KtV* Efficacy coefficient, *mg/dL* Milligram per decilitre, *IGF1* Growth factor similar to insulin type 1

Regarding PA, we observed an average value of 72.9% of the predicted. Peak torque of knee extensors had a predict average of 46.3 Nm. Average perfomance in 6MST was 71.2% of the predicted value for the studied sample. Individuals presented an average of 6.6 points in the MIS. For the HGS measurement, only three patients had venous artery fistula in the dominant limb, and thus the contralateral limb was evaluated. The results of body composition, peripheral muscle function and exercise tolerance are shown in Table 2.

**Table 2:** Results of body composition, peripheral muscle function and exercise tolerance

**Body Composition**	
Phase Angle (degrees)	4.2 ± 1.3
Phase Angle (% predicted)	72.9 ± 15.8
Malnutrition and inflammation score (points)	6.6 ± 3.2
**Peripheral muscle function**	
Peak Torque DLL (Nm)	66.9 ± 25.1
Peak Torque DLL (% predicted)	46.3 ± 10.9
Fatigue index RLL (%)	18.3 ± 21.9
Fatigue index LLL (%)	14.2 ± 17.8
Handgrip Strength (Kgf)	25.6 ± 9.4
Handgrip Strength (% predicted)	74.0 ± 26.5
Medical Council Research (points)	53.0 ± 6.2
**Exercise tolerance**	
6MST (steps)	71.6 ± 49.4
6MST (% predicted)	51.6 ± 33.0
**Quality of life**	
KQDOL (points)	98.6 ± 97

Notes: Data are presented as mean ± SD. *Abbreviations*: *DLL* Dominant lower limb, *Nm* Newton, *RLL* Right lower limb, *LLL* Left lower limb, *Kgf* Kilogram-force, *6MST* Six-minute step test, *KQDOL* Kidney Disease Quality of Life short form

### Correlation between phase angle, peak torque of knee extensors, the perfomance in the 6MST and the score in quality of life questionnaire

**Fig. 2:**
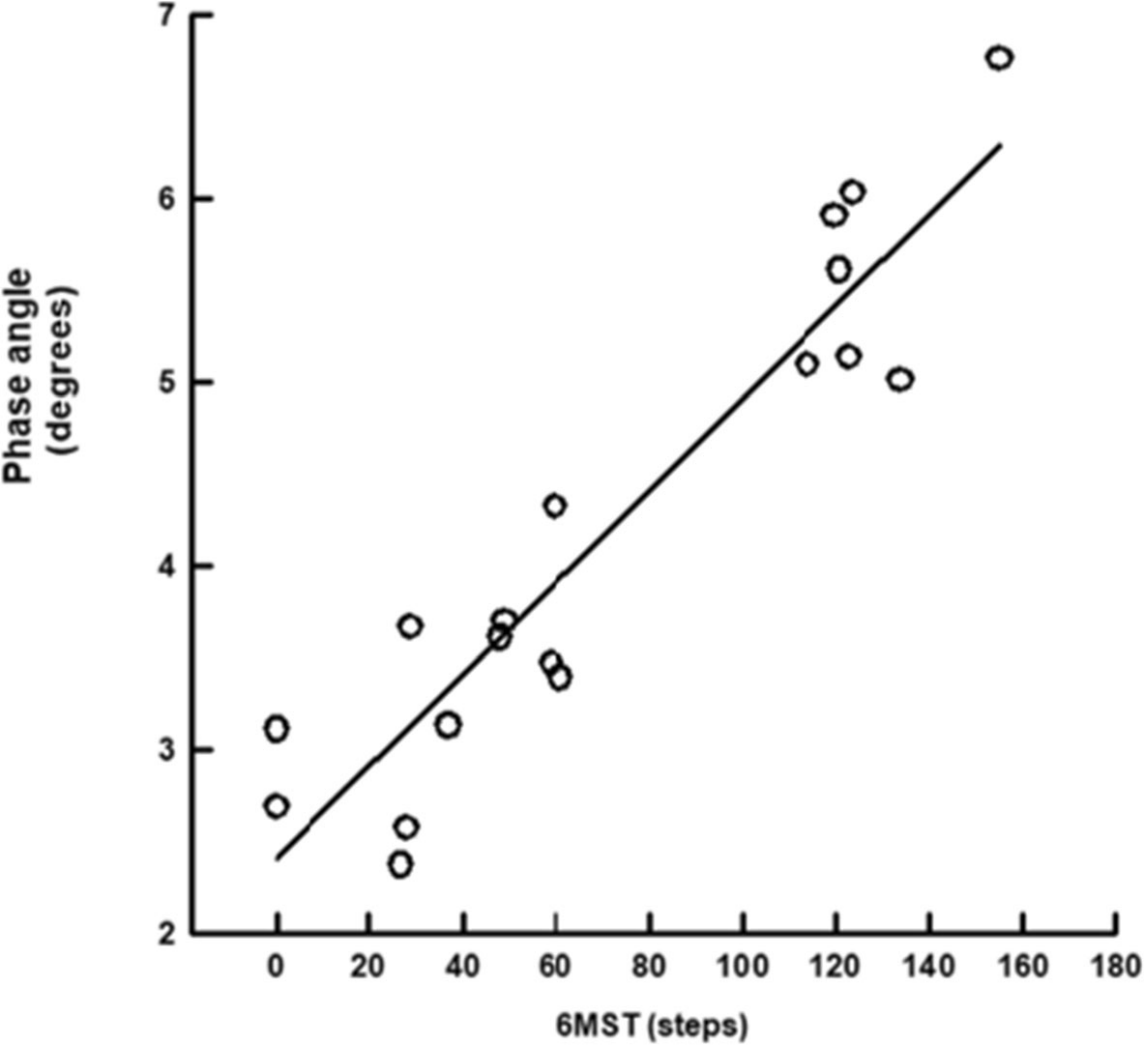
Linear regression curve of phase angle and the performance in 6MST. Abbreviations: 6MST, six-minute step test

The linear regression curve between PA and the performance in 6MST is shown in Fig. 2. There was a positive linear correlation between both, PA = (2.405 + 0.0251) x 6MST (r = 0.93, *p* < .001).

**Fig. 3:**
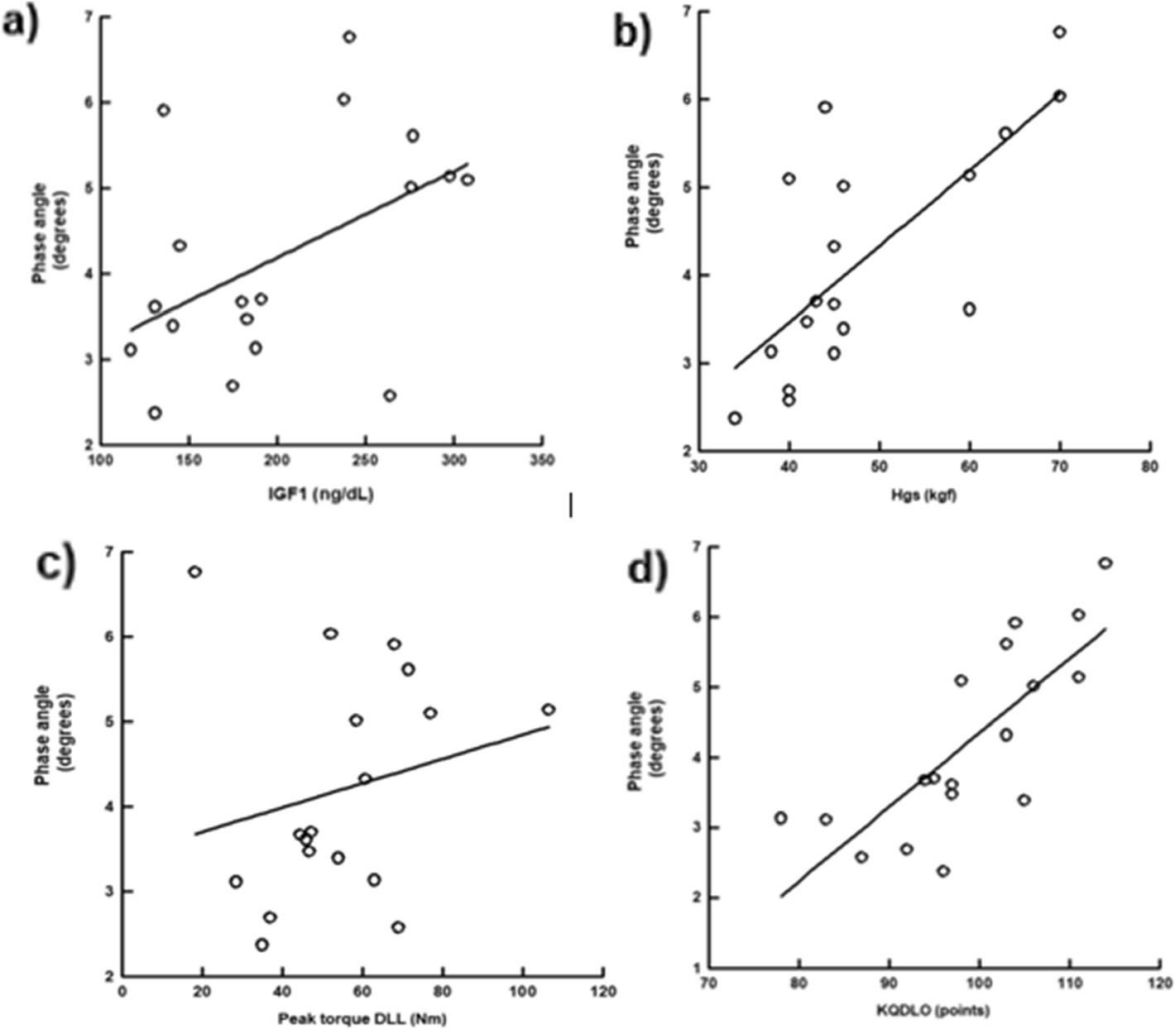
Linear regression curve between phase angle and handgrip strength, torque peak of knee extensors, IGF1, and quality of life questionnaire. Abbreviations: kgf, kilogram/force; DLL, dominant lower limb; Nm, Newton; IGF1, growth factor similar to insulin type 1; mg/dL, milligram per decilitre; KQDOL, kidney disease quality of lif short form

In the same sense, PA has a positive linear correlation with IGF1, PA = (2.163 + 0.0101) x IGF1 (r = 0.48, *p* < .001) (Fig. 3a); with HGS, PA = (0.0124 + 0.0864) x HGS (r = 0.72, *p* < .001) (Fig. 3b); with peak torque of knee extensors, PA = (3.415 + 0.0143) x peak torque of knee extensors (r = 0.21, *p* < .001) (Fig. 3c); and a negative linear correlation with KQDOL-SF, PA = (− 6.211 + 0.106) x KQDOL-FS (r = 0.77, *p* < .001) (Fig. 3d).

### Correlation between peak torque of knee extensors, malnutrition-inflammation score, IGF-1, and the performance in six-minute step test

**Fig. 4:**
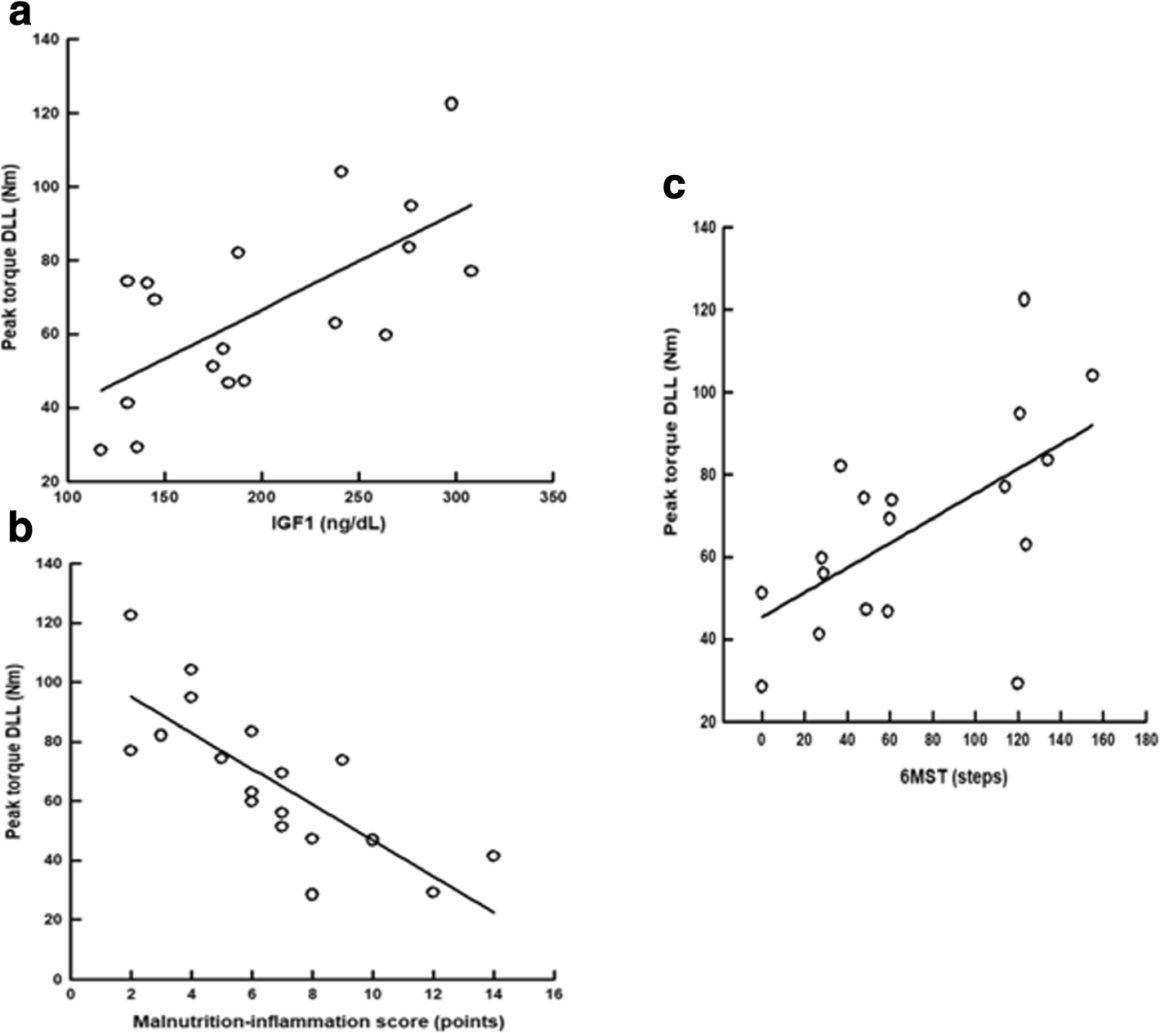
Linear regression curve of peak torque of knee extensors with MIS, IGF1, and performance in 6MST. Abbreviations: DLL, dominant lower limb; Nm, Newton; IGF1, growth factor similar to insulin type 1; mg/dL, milligram per decilitre; 6MST, six-minute step test

We also found a positive linear correlation between peak torque of knee extensors and IGF1, peak torque of knee extensors = (13.914 + 0.263) x IGF1 (r = 0.66, *p* < .001) (Fig. 4a); a negative linear correlation between peak torque of knee extensors and MIS, peak torque of knee extensors = (107.184–6.044) x MIS (r = − 0.77, *p* < .001) (Fig. 4b); and a positive linear correlation between peak torque of knee extensors and the performance in 6MST, peak torque of knee extensors = (45.391 + 0.300) x 6MST (r = 0.59, *p* < .001) (Fig. 4c).

## Discussion

We found a positive correlation between PA and the perfomance in 6MST. PA reflects the integrity of the cell membrane, and appears to be directly linked to healthy muscle tissues capable of producing energy [42, 43], thus, being able to explain the high correlation that founded. Some previous studies have evaluated the correlation of PA, mainly with mobility tests and evaluation by accelerometer, and few others have used 6MWT [44]. The present study, however, was a pioneer in evaluating exercise tolerance using 6MST and showing its correlation with PA.

The 6MST has been studied since the 1920s [46], and it has proved to be low cost and easy to apply, requiring only one step for its performance. This test activates a greater number of muscle groups, offering an accurate assessment of exercise tolerance specially in patients with chronic disease, and it seems to promote a cardiorespiratory demand closer to that a cardiopulmonary test when compared to other field tests, like 6MWT [45]. Besides that, 6MWT requires a 30-m corridor with the interruption of the flow of people at the test local, the 6MST does not require ample physical space, making its execution more feasible, especially in the context of hospital and clinical environments [17].

Patients with CKD commonly have PA values below the predicted, and the findings in this study are similar to those in the literature, as we observed that the average percentage of the predicted values of PA was 72.9%. PA has also been used in several populations, such as critical patients, where PA was a predictor of severity, mortality and length of hospital stay [47]; also in patients with lung cancer, where PA was associated with mortality and poor prognosis [48]. In another study, the PA was a survival predictor, in patients undergoing peritoneal HD [49].

The correlation found between PA with HGS and peak torque of knee extensors can also be explained based on the principle that PA is considered a predictor of body cell mass, which is mostly composed of intracellular water, and skeletal muscle tissue is composed of approximately 75% of water [49].. Therefore, it is considered that the higher the PA, the greater the quantity and quality of muscle mass [50].

The same thought applies to the correlations observed between PA and IGF1, once the roles played by IGF1 in muscle syntheses are already established, this trophism muscle marker acts in the growth and development of muscle fiber [51, 52].

The exercise tolerance in this population is known to be reduced, and our data also showed similar results, where the average performance of the 6MWT was below of the predicted [53]. Other authors described in their findings that the exercise tolerance impacted not only activities of daily living, but also cognitive function [54].

Likewise, another study that followed patients on HD for approximately 7 years, and found that low serum levels of nutritional markers such as albumin, reduced functional capacity, and reduced peripheral muscle strength were predictors of mortality in the evaluated individuals [55].

Inflammatory and nutritional status seems to directly affect peripheral muscle function. Additionally, there is a reduction in food intake and an increase in gastrointestinal disorders; an uncontrolled hydric balance; an anaerobic activity which can increase serum levels of lactate; metabolic acidosis; and reduction in the levels of ferritin and albumin. Moreover, there is also a reduction in the syntheses of muscle fibers, atrophy, increased proteolysis and energy expenditure, and concentration of inflammatory cytokines, leading to reduction of the quality and quantity of skeletal muscle tissue, directly impacting peripheral muscle function, as we also observed in our results [56, 57].

The present study has some limitations. This study was carried out in a single center and therefore having a reduced number of individuals composing the sample, could influence the extrapolation of the findings to others HD center, representing a limitation. The large number of male individuals (77% of sample) in one of them. However, the prevalence of men is high in the HD Centre of the Sírio-Libanês Hospital, where the data were collected, as well as in the Brazilian Dialysis Census, which could explain this distribution.

Another limitation is that it was not possible to control factors such as fasting before electrical bioimpedance, but we believe that this population has a rigorous routine including food intake schedules, reducing the chances of impact on evaluations. Likewise, individuals were not subdivided into sub categories for statistical analysis, wich may represent a limitation.

## Practical applications

The findings of this study reinforce the importance of continuous assessment of body composition, peripheral muscle function and exercise tolerance in HD patients. The 6MST is a feasible tool for assessing exercise tolerance in this population, especially in settings such as HD centers and hospitals.

Moreover, the PA appears to be a strong marker of exercise tolerance in this population. Our results show that the use of PA could be an option for initial screening in environments where there is no physical therapist available, to refer these individuals early to a rehabilitation program.

## Conclusion

We conclude that the PA and peak torque of knee extensors have a correlation with the performance in 6MST in patients with CKD undergoing HD. Therefore, PA and peak torque of knee extensors can be used for indirect evaluation of the 6MST in clinical practice. We suggest that future studies assess the cutoff point of PA to determine the reduced exercise tolerance.

## Supplementary Information

**Additional file 1.**

## Data Availability

The datasets used and/or analyzed during the current study available from the corresponding author on reasonable request.
