# Supplementary material for: Relationship of phase angle and peak torque of knee extensors with the performance in six-minute step test in haemodialysis patients
Source: BMC Nephrol. 2021 Feb 8;22:56. doi: 10.1186/s12882-021-02256-7 (PMC7869460; doi:10.1186/s12882-021-02256-7)
Supplement: Supplementary file 1 — Additional file 1. [file 12882_2021_2256_MOESM1_ESM.pdf]

# RELATIONSHIP OF PHASE ANGLE AND PEAK TORQUE OF KNEE EXTENSORS WITH THE PERFORMANCE IN SIX-MINUTE STEP TEST IN HAEMODIALYSIS PATIENTS.

Camila Porto Brito[1], Igor Gutierrez Moraes[1], Cláudio Luders[1], Christina May Moran de Brito[1], Wellington Pereira Yamaguti[1]

[1]Hospital Sírio-Libanês

Corresponding author: Wellington Pereira Yamaguti Hospital Sírio-Libanês, Rehabilitation Centre, Dona Adma Jafet Street, 91 – Bela Vista, São Paulo/SP, Brazil, 01308-050 Telephone Number +55 11 97286 0465 Email wellington.psyamaguti@hsl.org.br

# ***DATA COLLECTION FORM***

## *1. PERSONAL AND HEALTH HISTORY*

**Patient Code:___________ Date _____/____/_____**

**Personal Data**

**Age group: ( ) I 18-30 ( ) II 30-40 ( ) III 40- 50 ( ) IV 50- 60 ( ) V 60-70 ( ) VI 70-80 ( ) VII >80**

**Name: ______________________________________________________________________**

**Sex ( ) F ( ) M Birth Date: ___/___/___ Age:____Ethnicity:________Marital Status: _______________**

**Ocuppation:____________________( years) Previous Ocuppation: _____________________________**

**Scholarity: ( ) college complete ( ) incomplete college ( ) complete high school ( ) incomplete high school ( ) literate ( ) not literate**

**Adress: ____________________________________________________________________**

**Postal code: ___________-______ District: _______________ City: ( ) São Paulo ( ) Other: _______________**

**Phone number: ( ) ______________( ) __________Email: ________________________________________**

**1.a) Life habits**

**( ) Smoker ( ) Ex-smoker Smoking load: ________**

**( ) Drink alcohol Weekly frequency: _________**

**( ) Sedentary ( ) Physical activity ________________________ Weekly frequency: _______**

**Physician responsible: _______________________________________________________**

**1.b) Personal Background**

**Diseases:**

**( ) systemic arterial hypertension ( ) systemic arterial hypotension ( ) Diabetes mellitus ( ) Hypothyroidism ( ) Dyslipidemia ( ) Stroke ( ) Coronary insufficiency ( ) acute myocardial infarction ( ) Bronchopneumonia ( ) Tuberculosis ( ) COPD ( )Asthma ( ) Rinithis ( ) Sinusitis ( ) Obstructive sleep apnea syndrom**

**Etiology of kidney disease: ______________________________________________________________**

**Hemodialysis frequency:** ______________________________________________________

**Hemodialysis session duration:** __________________________________________________________

**Other diseases:** ________________________________________________________________________

**( ) Allergies** ___________________________________________________________________________

**( ) Osteomioarticular diseases:** ___________________________________________________________

**Previous surgeries :** _____________________________________________________________________

**( ) Previous hospitalization**

**Date of last hospital stay?** ______________________________________________________________

**Reason of last hospital stay:** ______________________________________________________________

**Time of last hospital stay:**______________**Outcome:** __________________________________________

**Medications in use/ Dosage**

| | |
|---|---|
| | |
| | |
| | |
| | |
| | |

**1.c) Anthropometric Date**

**Date (___/___/___)** **Evaluator:** __________________________

**Weight:** _____ **Height:** _____ **m** **BMI:** ________

**Classification: ( ) low weight ( ) normal ( ) overweight ( ) obesity**

## 2. MEDICAL RESEARCH COUNCIL

| Medical Research Council (MRC) | Right side | Left side | Total |
|---|---|---|---|
| Shoulder abduction | | | |
| Elbow flexion | | | |
| Wrist extension | | | |
| Hip flexion | | | |
| Knee extension | | | |
| Ankle dorsiflexion | | | |

**Total Score** [ ]

## 3.HANDGRIP STRENGTH

| HANDGRIP STRENGTH | 1st measure | 2nd measure | 3rd measure |
|---|---|---|---|
| Score | | | |

**Dominant Side**

**( ) R ( ) L**

# 4. SIX-MINUTE STEP TEST

| Six Minute Step Test | | | | | | | |
|---|---|---|---|---|---|---|---|
| Time | Heart Hate | Respiratory frequency | Blood pessure | Peripheral Oxygen Saturation | BORG (lower limb) | BORG (dyspnea) | STEPS |
| Rest | | | | | | | |
| 1st min | | | | | | | |
| 2nd min. | | | | | | | |
| 3rd min. | | | | | | | |
| 4th min. | | | | | | | |
| 5th min. | | | | | | | |
| 6th min. (FINAL) | | | | | | | |
| 2nd min after the end of the test (Recovery) | | | | | | | |

**Stopped during the test? Yes ( ) No ( ) Reason:**______________________________________________

**( ) ventilatory ( ) cardiac ( ) peripheral muscle ( ) Others** _________________________

**Total time stopped during the test:**________________________________

**Adverse event:** _________________________________________________________________

# ***5. QUESTIONNAIRE 1: MALNUTRITION AND INFLAMMATION SCORE (MIS)***

**(A).Medical History**

1.Dry weight change (after hemodialysis session) in the last three to six months?

☐ Without weight reduction or reduction less than 0.5kg ( 0 point )

☐ Weight reduction greater than 0.5kg and less than 1kg ( 1 point )

☐ Weight reduction greater than 1 kg and less than 5% of body weight ( 2 points )

☐ Weight reduction greater than 5% of body weight ( 3 points )

2. Food Intake

☐ Good food intake and without worsening food pattern ( 0 point )

☐ Ingestion of solid but sub-optimal diet (1 point )

☐ Just liquid diet ( 2 points )

☐ Hypocaloric liquid diet or fasting ( 3 points )

3. Gastrointestinal symptons

☐ No symptons with good food intake ( 0 point )

☐ Mild symptoms, poor food intake, occasional nausea ( 1 point )

☐ Moderated symptoms with frequent nausea ( 2 points )

☐ Frequent diarrhea, vomiting or severe anorexia ( 3 points )

4. Functional capacity

☐ Normal or improved functional capacity ( 0 point )

☐ Occasional difficulty walking or feeling tired frequently ( 1 point )

☐ Difficulty performing tasks that used to do without help ( 2 points )

☐ Restricted to bed or chair, with little or no physical activity ( 3 points )

5. Comorbities including the number of years undergoing hemodialysis

☐ On hemodialysis for less than one year and feeling well ( 0 point )

☐ On hemodialysis between one and four years, or presence of mild comorbidities, without MC* ( 1 point )

☐ On hemodialysis for more than four years or with moderate comorbidities with MC* ( 2 points )

☐ Any severe or multiple comorbidity with two or more MC* ( 3 points )

*MC: Main Comorbidities: include chronic heart failure (functional class III and IV), AIDS, severe coronary insufficiency, moderate to severe COPD, severe neurological sequelae, metastatic cancer and recent chemotherapy.

**(B) Physical Exam**

6. Decreased body fat reserve

☐ Normal ( 0 point )

☐ Slight ( 1 point )

☐ Mild ( 2 points )

☐ Severe ( 3 points )

7. Signs of reduced muscle mass

☐ Normal ( 0 point )

☐ Slight ( 1 point )

☐ Mild ( 2 points )

☐ Severe ( 3 points )

8. Body Mass Index (BMI)

☐ Greater or equal to 25kg/$m^2$ ( 0 point )

☐ Between 18 e 19,99kg/$m^2$( 1 point )

☐ Between 16 e 17,99kg/$m^2$ ( 2 points )

☐ Less than 16kg/$m^2$ ( 3 points )

9. Serum Albumin

☐ Greater or equal to 4,0g/dL ( 0 point )

☐ Between 3,5 e 3,9g/dL ( 1 point )

☐ Between 3,0 e 3, 4g/dL ( 2 points )

☐ Less than 3,0g/dL ( 3 points )

10. Ferritin

☐ Greater or equal to 250 mg/dL ( 0 point )

☐ Between 200 e 249mg/dL ( 1 point )

☐ Between 150 e 199mg/dL ( 2 points )

☐ Less than 150 mg/dL ( 3 points )

**TOTAL SCORE**: ☐

## ***6. QUESTIONNAIRE 2: QUALITY OF LIFE QUESTIONNAIRE – (KDQOL-SF)***

**1. In general, would you say your health is:**

| Excellent | Very good | Good | Fair | Poor |
|---|---|---|---|---|
| ☐ 1 | ☐ 2 | ☐ 3 | ☐ 4 | ☐ 5 |

**2.Compared to one year ago, how would you rate your health in general now?**

| Much better now than one year ago | Somewhat better now than one year ago | About the same as one year ago | Somewhat worse now than one year ago | Much worse now than one year ago |
|---|---|---|---|---|

☐ 1 ☐ 2 ☐ 3 ☐ 4 ☐ 5

**3. The following items are about activities you might day. Does your health now limit you in these activities? If so, how much?**

| | | Yes, limited a lot | Yes, limited a little | No, not limited at all |
|---|---|---|---|---|
| a. | Vigorous activities, such as running, lifting heavy objects, participating in strenuous sports | ☐ 1 | ☐ 2 | ☐ 3 |
| b. | Moderate activities, such as moving a table, pushing a vacuum cleaner, bowling, or playing golf | ☐ 1 | ☐ 2 | ☐ 3 |
| c. | Lifting or carrying groceries | ☐ 1 | ☐ 2 | ☐ 3 |
| d. | Climbing several flights of stairs | ☐ 1 | ☐ 2 | ☐ 3 |
| e. | Climbing one flight of stairs | ☐ 1 | ☐ 2 | ☐ 3 |
| f. | Bending, kneeling, or stooping | .☐ 1 | ☐ 2 | ☐ 3 |
| g. | Walking more than a mile | .☐ 1 | ☐ 2 | ☐ 3 |
| h. | Walking 500 meters | .☐ 1 | ☐ 2 | ☐ 3 |
| i. | Walking 100 meters | .☐ 1 | ☐ 2 | ☐ 3 |
| j. | Bathing or dressing yourself | ☐ 1 | ☐ 2 | ☐ 3 |

**4. During the past 4 weeks, have you had any of the following problems with your work or other regular daily activities as a result of your physical health?**

| | | Yes | No |
|---|---|---|---|
| a | Cut down on the amount of time you spent on work or other activities? | ☐ 1 | ☐ 2 |
| b | Accomplished less than you would have liked? | ☐ 1 | ☐ 2 |
| c | Were limited in the kind of work or other activities? | ☐ 1 | ☐ 2 |
| d | Had difficulty performing the work or other activities (for example, it took extra effort)? | ☐ 1 | ☐ 2 |

**5. During the past 4 weeks, have you had any of the following problems with your work or other regular daily activities as a result of any emotional problems (such as feeling depressed or anxious)?**

| | | Yes | No |
|---|---|---|---|
| a | Cut down on the amount of time you spent on work or other activities? | ☐ 1 | ☐ 2 |
| b | Accomplished less than you would like? | ☐ 1 | .☐ 2 |
| c | Didn't do work or other activities as carefully as usual? | ☐ 1 | .☐ 2 |

**6. During the past 4 weeks, to what extent has your physical health or emotional problems interfered with your normal social activities with family, friends, neighbours, or clubs?**

| Not at all | Slightly | Moderately | Quite a bit | Extremely |
|---|---|---|---|---|
| ☐ 1 | ☐ 2 | ☐ 3 | ☐ 4 | ☐ 5 |

**7. How much bodily pain have you had during the past 4 weeks?**

| None | Very mild | Mild | Moderate | Severe | Very severe |
|---|---|---|---|---|---|
| ☐ 1 | ☐ 2 | ☐ 3 | ☐ 4 | ☐ 5 | ☐ 6 |

**8. During the past 4 weeks, how much did pain interfere with your normal work (including both work outside the home and housework)?**

| Not at all | A little bit | Moderately | Quite a bit | Extremely |
|---|---|---|---|---|
| ☐ 1 | ☐ 2 | ☐ 3 | ☐ 4 | ☐ 5 |

**9. These questions are about how you feel and how things have been with you during the past 4 weeks. For each question, please give the one answer that comes closest to the way you have been feeling.**

| | | All of the time | Most of the time | A good bit of the time | Some of the time | A little of the time | None of the time |
|---|---|---|---|---|---|---|---|
| a. | Did you feel full of life? | ☐ 1 | ☐ 2 | ☐ 3 | ☐ 4 | ☐ 5 | ☐ 6 |
| b | Have you been a very nervous person? | ☐ 1 | ☐ 2 | ☐ 3 | ☐ 4 | ☐ 5 | ☐ 6 |
| c | Have you felt so down in the dumps that nothing could cheer you up? | ☐ 1 | ☐ 2 | ☐ 3 | ☐ 4 | ☐ 5 | ☐ 6 |
| d | Have you felt calm and peaceful? | ☐ 1 | ☐ 2 | ☐ 3 | ☐ 4 | ☐ 5 | ☐ 6 |
| e | Did you have a lot of energy? | ☐ 1 | ☐ 2 | ☐ 3 | ☐ 4 | ☐ 5 | ☐ 6 |
| f | Have you felt downhearted and unhappy? | ☐ 1 | ☐ 2 | ☐ 3 | ☐ 4 | ☐ 5 | ☐ 6 |
| g | Did you feel worn out? | ☐ 1 | ☐ 2 | ☐ 3 | ☐ 4 | ☐ 5 | ☐ 6 |
| h | Have you been a happy person? | ☐ 1 | ☐ 2 | ☐ 3 | ☐ 4 | ☐ 5 | ☐ 6 |
| i | Did you feel tired? | ☐ 1 | ☐ 2 | ☐ 3 | ☐ 4 | ☐ 5 | ☐ 6 |

**10. During the past 4 weeks, how much of the time has your physical health or emotional problems interfered with your social activities (like visiting with friends, relatives, etc.)?**

| All of the time | Most of the time | Some of the time | A little of the time | None of the time |
|---|---|---|---|---|
| ☐ 1 | ☐ 2 | ☐ 3 | ☐ 4 | ☐ 5 |

**11. Please choose the answer that best describes how true or false each of the following statements is for you.**

| | | Definitely true | Mostly true | Don't know | Mostly false | Definitely false |
|---|---|---|---|---|---|---|
| a | I seem to catch things a little more easily than other people | ☐ 1 | ☐ 2 | ☐ 3 | ☐ 4 | ☐ 5 |
| b | I am as healthy as anybody I know | ☐ 1 | ☐ 2 | ☐ 3 | ☐ 4 | ☐ 5 |
| c | I expect my health to get worse | ☐ 1 | ☐ 2 | ☐ 3 | ☐ 4 | ☐ 5 |
| d | My health is excellent | ☐ 1 | ☐ 2 | ☐ 3 | ☐ 4 | ☐ 5 |

**12. How true or false is each of the following statements for you?**

| | | Definitely true | Mostly true | Don't know | Mostly false | Definitely false |
|---|---|---|---|---|---|---|
| a | My kidney disease interferes too much with my life | ☐ 1 | ☐ 2 | ☐ 3 | ☐ 4 | ☐ 5 |
| b | Too much of my time is spent dealing with my kidney disease | ☐ 1 | ☐ 2 | ☐ 3 | ☐ 4 | ☐ 5 |
| c | I feel frustrated dealing with my kidney disease | ☐ 1 | ☐ 2 | ☐ 3 | ☐ 4 | ☐ 5 |
| d | I feel like a burden on my family | ☐ 1 | ☐ 2 | ☐ 3 | ☐ 4 | ☐ 5 |

**13. These questions are about how you feel and how things have been going during the past 4 weeks. For each question, please give the one answer that comes closest to the way you have been feeling.**

| | | None of the time | A little of the time | Some of the time | A good bit of the time | Most of the time | All of the time |
|---|---|---|---|---|---|---|---|
| a | Did you isolate yourself from people around you? | ☐ 1 | ☐ 2 | ☐ 3 | ☐ 4 | ☐ 5 | ☐ 6 |
| b | Did you react slowly to things that were said or done? | ☐ 1 | ☐ 2 | ☐ 3 | ☐ 4 | ☐ 5 | ☐ 6 |
| c | Did you act irritable toward those around you? | ☐ 1 | ☐ 2 | ☐ 3 | ☐ 4 | ☐ 5 | ☐ 6 |
| d | Did you have difficulty concentrating or thinking? | ☐ 1 | ☐ 2 | ☐ 3 | ☐ 4 | ☐ 5 | ☐ 6 |
| e | Did you get along well with other people? | ☐ 1 | ☐ 2 | ☐ 3 | ☐ 4 | ☐ 5 | ☐ 6 |
| f | Did you become confused? | ☐ 1 | ☐ 2 | ☐ 3 | ☐ 4 | ☐ 5 | ☐ 6 |

**14. During the past 4 weeks, to what extent were you bothered by each of the following?**

| | | Not at all bothered | Somewhat bothered | Moderately bothered | Very much bothered | Extremely bothered |
|---|---|---|---|---|---|---|
| a | Soreness in your muscles? | ☐ 1 | ☐ 2 | ☐ 3 | ☐ 4 | ☐ 5 |
| b | Chest pain? | ☐ 1 | ☐ 2 | ☐ 3 | ☐ 4 | ☐ 5 |
| c | Cramps? | ☐ 1 | ☐ 2 | ☐ 3 | ☐ 4 | ☐ 5 |
| d | Itchy skin? | ☐ 1 | ☐ 2 | ☐ 3 | ☐ 4 | ☐ 5 |
| e | Dry skin? | ☐ 1 | ☐ 2 | ☐ 3 | ☐ 4 | ☐ 5 |
| f | Shortness of breath? | ☐ 1 | ☐ 2 | ☐ 3 | ☐ 4 | ☐ 5 |
| g | Faintness or dizziness? | ☐ 1 | ☐ 2 | ☐ 3 | ☐ 4 | ☐ 5 |
| h | Lack of appetite? | ☐ 1 | ☐ 2 | ☐ 3 | ☐ 4 | ☐ 5 |
| i | Washed out or drained? | ☐ 1 | ☐ 2 | ☐ 3 | ☐ 4 | ☐ 5 |
| j | Numbness in hands or feet? | ☐ 1 | ☐ 2 | ☐ 3 | ☐ 4 | ☐ 5 |
| k | Nausea or upset stomach? | ☐ 1 | ☐ 2 | ☐ 3 | ☐ 4 | ☐ 5 |
| | Problems with your access site? | ☐ 1 | ☐ 2 | ☐ 3 | ☐ 4 | ☐ 5 |

**15. Some people are bothered by the effects of kidney disease on their daily life, while others are not. How much does kidney disease bother you in each of the following areas?**

| | | Not at all bothered | Somewhat bothered | Moderately bothered | Very much bothered | Extremely bothered |
|---|---|---|---|---|---|---|
| a | Fluid restriction? | ☐ 1 | ☐ 2 | ☐ 3 | ☐ 4 | ☐ 5 |
| b | Dietary restriction? | ☐ 1 | ☐ 2 | ☐ 3 | ☐ 4 | ☐ 5 |
| c | Your ability to work around the house? | ☐ 1 | ☐ 2 | ☐ 3 | ☐ 4 | ☐ 5 |
| d | Your ability to travel? | ☐ 1 | ☐ 2 | ☐ 3 | ☐ 4 | ☐ 5 |
| e | Being dependent on doctors and other medical staff? | ☐ 1 | ☐ 2 | ☐ 3 | ☐ 4 | ☐ 5 |
| f | Stress or worries caused by kidney disease? | ☐ 1 | ☐ 2 | ☐ 3 | ☐ 4 | ☐ 5 |
| g | Your sex life? | ☐ 1 | ☐ 2 | ☐ 3 | ☐ 4 | ☐ 5 |
| h | Your personal appearance? | ☐ 1 | ☐ 2 | ☐ 3 | ☐ 4 | ☐ 5 |

**16. The next two questions are personal and relate to your sexual activity, but your answers are important in understanding how kidney disease impacts on people's lives. How much of a problem was each of the following in the past 4 weeks?**

| | | Not a problem | A little problem | Somewhat of a problem | Very much a problem | Severe problem |
|---|---|---|---|---|---|---|
| a | Enjoying sex? | ☐ 1 | ☐ 2 | ☐ 3 | ☐ 4 | ☐ 5 |
| b | Becoming sexually aroused? | ☐ 1 | ☐ 2 | ☐ 3 | ☐ 4 | ☐ 5 |

**17. For the following question, please rate your sleep using a scale ranging from 0 representing "very bad" to 10 representing "very good". On a scale from 0 to 10, how would you rate your sleep overall?**

Very bad — Very good

| 0 | 1 | 2 | 3 | 4 | 5 | 6 | 7 | 8 | 9 | 10 |
|---|---|---|---|---|---|---|---|---|---|---|
| ☐ | ☐ | ☐ | ☐ | ☐ | ☐ | ☐ | ☐ | ☐ | ☐ | ☐ |

**18. How often during the past 4 weeks did you...**

| | | None of the time | A Little of the time | Some of the time | A good bit of the time | Most of the time | All of the time |
|---|---|---|---|---|---|---|---|
| a | Awaken during the night and have trouble falling asleep again? | ☐ 1 | ☐ 2 | ☐ 3 | ☐ 4 | ☐ 5 | ☐ 6 |
| b | Get the amount of sleep you need? | ☐ 1 | ☐ 2 | ☐ 3 | ☐ 4 | ☐ 5 | ☐ 6 |
| c | Have trouble staying awake during the day? | ☐ 1 | ☐ 2 | .☐ 3 | ☐ 4 | ☐ 5 | ☐ 6 |

**19. Concerning your family and friends, how satisfied are you with...**

| | | Very dissatisfied | Somewhat dissatisfied | Somewhat satisfied | Very satisfied |
|---|---|---|---|---|---|
| a | The amount of time you are able to spend with your family and friends? | ☐ 1 | ☐ 2 | ☐ 3 | ☐ 4 |
| b | The support you receive from your family and friends? | ☐ 1 | ☐ 2 | .☐ 3 | .☐ 4 |

**20. During the past 4 weeks, did you work at a paying job?**

| Yes | No |
|---|---|
| ☐ 1 | ☐ 2 |

**21. Does your health keep you from working at a paying job?**

| Yes | No |
|---|---|
| ☐ 1 | ☐ 2 |

**22. Overall, how would you rate your health?**

| Worst possible (as bad or worse than being dead) | | | | | Half-way between worst and best | | | | | Best possible health |
|---|---|---|---|---|---|---|---|---|---|---|
| 0 | 1 | 2 | 3 | 4 | 5 | 6 | 7 | 8 | 9 | 10 |
| ☐ | ☐ | ☐ | ☐ | ☐ | ☐ | ☐ | ☐ | ☐ | ☐ | ☐ |

**23. Think about the care you receive for kidney dialysis. In terms of your satisfaction, how would you rate the friendliness and interest shown in you as a person?**

| Very poor | Poor | Fair | Good | Very good | Excellent | The Best |
|---|---|---|---|---|---|---|
| ☐ 1 | ☐ 2 | ☐ 3 | ☐ 4 | ☐ 5 | ☐ 6 | ☐ 7 |

**24. How true or false is each of the following statements?**

| | | Definitely true | Mostly true | Don't know | Mostly false | Definitely false |
|---|---|---|---|---|---|---|
| a | Dialysis staff encourage me to be as independent as possible | ☐ 1 | ☐ 2 | ☐ 3 | ☐ 4 | ☐ 5 |
| b | Dialysis staff support me in coping with my kidney disease | ☐ 1 | ☐ 2 | ☐ 3 | ☐ 4 | ☐ 5 |
